# Supplementary material for: A polymorphism in the cachexia-associated gene INHBA predicts efficacy of regorafenib in patients with refractory metastatic colorectal cancer
Source: PLoS One. 2020 Sep 24;15(9):e0239439. doi: 10.1371/journal.pone.0239439 (PMC7514061; doi:10.1371/journal.pone.0239439)
Supplement: S1 Table — (DOCX) [file pone.0239439.s001.docx]

**Supplemental Table 1. Polymorphisms and primers.**

| Gene | Location of polymorphism | Change | Function | MAF | Forward / reverse primer (5'–3') |
| --- | --- | --- | --- | --- | --- |
| ***INHBA*** | intron |  |  |  | F: TGTGATAGCCACAGCCTCAA |
| rs17776182 | Chr 7, 41718280 | G > A | Risk of invasive ovarian cancer | 0.16 | R: TTCCAAACCTCAGTGGCTTC |
| ***INHBA*** | intron |  |  |  | F: AAGATTTCACTGTGCCTAGATATGG |
| rs2237432 | Chr 7, 41695436 | A > G | Risk of infertility | 0.22 | R: AAGGAGGAATAACCTTAAGACCTCA |
| ***MSTN*** | intron |  |  |  | F: CATCAGCGGATGAATGGATA |
| rs7570532 | Chr 2, 190058686 | A > G | Risk of osteoporotic fracture | 0.36 | R: GCATAGCTTAGCTCGCACTTG |
| ***ALK4(ACVR1B)*** | 3' UTR |  |  |  | F: CTGGTGGAAGTCTTGGGTGT |
| rs2854464 | Chr12, 51995107 | A > G | Muscle strength | 0.37 | R: TCTGGGAGATGAAAGACAGATG |
| ***ALK5 (TGFBR1)*** | intron |  |  |  | F: CTTGCCTTACCATGGGAGAA |
| rs10760673 | Chr 9, 99116340 | G > A | Protein coding | 0.27 | R: ATTTCCTCAGGGACACACCA |
| ***ALK7(ACVR1C)*** |  |  | Transcriptional regulation |  | F: CCAGAGCTCACCATGTATCCT |
| rs13010956 | Chr 2, 157556030 | T > C | Metabolic syndrome | 0.33 | R: TTATGATGTGACCGCCTCTG |
| ***ACVR2B*** | intron |  | Transcriptional regulation |  | F: GGAGCTCAGGGTAGTGCAAA |
| rs2268753 | Chr 3, 38458698 | T > C | Associated with ovarian failure | 0.43 | R: GGACCCTGCCTCAGGACTAT |
| ***ACVR2B*** | 3' UTR |  |  |  | F: AAGACTGCCAGTGAGGGAAG |
| rs13072731 | Chr 3, 38491844 | C > A | Transcriptional regulation | 0.39 | R: GGCATTGTTGTGGATTTGTG |
| ***SMAD2*** | 3' UTR |  |  |  | F: TGGACACGATTATTCCGCAAAA |
| rs1792671 | Chr 18, 47835823 | C > T | Tag SNP | 0.47 | R: TGACCTTGTGATCCGCCTG |
| **SMAD2** | intron |  |  |  | F: GCCACAGAGAAAGGAAAACA |
| rs1792689 | Chr 18, 47842216 | G > A | Risk of rectal cancer | 0.13 | R: TACCCTCCAAACAGTTAACA |
| ***FOXO3*** | intron |  |  |  | F: TCAGTTGGGTTGGAATTGGT |
| rs12212067 | Chr 6, 108659993 | G > T | Associated with Crohn’s disease | 0.14 | R: CCCTCTGCGTTAGATTCTGG |
| ***FOXO3*** | 3' UTR |  |  |  | F: CTCAGTCCGGAAGTCTAGAACAG |
| rs4946935 | Chr 6, 108682118 | A > G | Increased life span | 0.48 | R: AAAATGCTCTGAAGTTGAAAAGC |

Abbreviations: 3’ UTR, 3’-untranslated region; Chr, chromosome; SNP, single-nucleotide polymorphism.

^a^Minor allele frequency (MAF), according to the Ensembl database (phase I of the 1000 Genomes Project) for Europeans.
