# Supplementary material for: A polymorphism in the cachexia-associated gene INHBA predicts efficacy of regorafenib in patients with refractory metastatic colorectal cancer
Source: PLoS One. 2020 Sep 24;15(9):e0239439. doi: 10.1371/journal.pone.0239439 (PMC7514061; doi:10.1371/journal.pone.0239439)
Supplement: S2 Table — (DOCX) [file pone.0239439.s002.docx]

**Supplemental Table 2. Association between cachexia-related gene polymorphism and clinical outcome in the validation cohort (Japanese regorafenib cohort).**

| Genotype | *N* | Disease control | | |  | Progression-free survival | | | | |  | Overall survival | | | | |
| --- | --- | --- | --- | --- | --- | --- | --- | --- | --- | --- | --- | --- | --- | --- | --- | --- |
|  |  | SD | PD | *P*  value* |  | Median, months (95%CI) | HR (95%CI) † | *P*  value* | HR (95%CI) ‡ | *P*  value* |  | Median, months (95%CI) | HR (95%CI) † | *P*  value* | HR (95%CI) ‡ | *P*  value* |
| ***INHBA* rs2237432** |  |  |  | 0.27 |  |  |  | 0.50 |  | 0.21 |  |  |  | 0.12 |  | **0.026** |
| A/A | 39 | 15 (45%) | 18 (55%) |  |  | 2.0 (1.8, 3.3) | 1 (Reference) |  | 1 (Reference) |  |  | 8.1 (5.0, 11.8) | 1 (Reference) |  | 1 (Reference) |  |
| A/G | 29 | 14 (61%) | 9 (39%) |  |  | 2.7 (1.8, 4.3) | 0.85 (0.52, 1.38) |  | 0.74 (0.45, 1.22) |  |  | 12.9 (6.3, 29.9+) | 0.57 (0.31, 1.07) |  | 0.48 (0.26, 0.91) |  |
| G/G | 9 | 2 (29%) | 5 (71%) |  |  | 1.3 (0.6, 2.4) | 1.27 (0.58, 2.78) |  | 1.49 (0.67, 3.31) |  |  | 4.0 (1.5, 26.2+) | 1.22 (0.50, 2.94) |  | 1.56 (0.63, 3.85) |  |
| ***MSTN* rs7570532** |  |  |  | 0.25 |  |  |  | 0.096 |  | 0.72 |  |  |  | 0.17 |  | 0.81 |
| A/A | 41 | 14 (44%) | 18 (56%) |  |  | 1.8 (1.7, 2.7) | 1 (Reference) |  | 1 (Reference) |  |  | 7.6 (4.6, 12.0) | 1 (Reference) |  | 1 (Reference) |  |
| A/G ^a^ | 30 | 12 (48%) | 13 (52%) |  |  | 2.3 (1.8, 3.7) | 0.69 (0.44, 1.10) |  | 0.91 (0.56, 1.49) |  |  | 11.8 (6.5, 26.7+) | 0.68 (0.39, 1.19) |  | 0.93 (0.52, 1.66) |  |
| G/G ^a^ | 6 | 5 (83%) | 1 (17%) |  |  |  |  |  |  |  |  |  |  |  |  |  |
| ***SMAD2* rs1792671** |  |  |  | 0.62 |  |  |  | 0.61 |  | 0.30 |  |  |  | 0.84 |  | 0.79 |
| G/G | 57 | 22 (50%) | 22 (50%) |  |  | 2.0 (1.7, 2.7) | 1 (Reference) |  | 1 (Reference) |  |  | 9.6 (5.9, 12.9) | 1 (Reference) |  | 1 (Reference) |  |
| G/A ^a^ | 18 | 9 (53%) | 8 (47%) |  |  | 2.5 (1.8, 4.3) | 0.88 (0.52, 1.48) |  | 0.76 (0.44, 1.29) |  |  | 7.9 (4.6, 15.3) | 1.06 (0.57, 1.97) |  | 0.92 (0.49, 1.71) |  |
| A/A ^a^ | 2 | 0 (0%) | 2 (100%) |  |  |  |  |  |  |  |  |  |  |  |  |  |
| ***FOXO3* rs12212067** |  |  |  | 0.18 |  |  |  | **0.027** |  | 0.26 |  |  |  | 0.054 |  | 0.20 |
| T/T | 63 | 21 (42%) | 29 (58%) |  |  | 1.8 (1.8, 2.3) | 1 (Reference) |  | 1 (Reference) |  |  | 7.6 (5.2, 10.3) | 1 (Reference) |  | 1 (Reference) |  |
| T/G ^a^ | 16 | 9 (64%) | 5 (36%) |  |  | 3.3 (1.9, 7.2) | 0.56 (0.32, 0.97) |  | 0.71 (0.39, 1.29) |  |  | 15.3 (3.6, 27.2) | 0.49 (0.23, 1.04) |  | 0.61 (0.28, 1.30) |  |
| G/G ^a^ | 1 | 1 (100%) | 0 (0%) |  |  |  |  |  |  |  |  |  |  |  |  |  |
| ***FOXO3* rs4946935** |  |  |  | 0.29 |  |  |  | 0.096 |  | 0.41 |  |  |  | 0.10 |  | 0.34 |
| G/G | 49 | 17 (43%) | 23 (58%) |  |  | 2.0 (1.8, 2.3) | 1 (Reference) |  | 1 (Reference) |  |  | 7.1 (5.0, 10.3) | 1 (Reference) |  | 1 (Reference) |  |
| G/A ^a^ | 24 | 10 (50%) | 10 (50%) |  |  | 2.5 (1.8, 4.5) | 0.69 (0.43, 1.11) |  | 0.81 (0.50, 1.33) |  |  | 12.6 (8.1, 27.2) | 0.62 (0.35, 1.12) |  | 0.75 (0.42, 1.35) |  |
| A/A ^a^ | 6 | 4 (80%) | 1 (20%) |  |  |  |  |  |  |  |  |  |  |  |  |  |

Abbreviations: SD, stable disease; PD, progressive disease; HR, hazard ratio; CI, confidence interval.

* *P* value based on Fisher’s exact test for tumor response, log-rank test for PFS and OS in the univariate analysis (†), and Wald test for PFS and OS in the multivariable Cox regression model adjusted for liver metastasis and lymph node involvement (‡). P values < 0.050 are shown in bold text.

^a^ In the dominant model.

+ Estimates not yet reached.
