# Supplementary material for: A polymorphism in the cachexia-associated gene INHBA predicts efficacy of regorafenib in patients with refractory metastatic colorectal cancer
Source: PLoS One. 2020 Sep 24;15(9):e0239439. doi: 10.1371/journal.pone.0239439 (PMC7514061; doi:10.1371/journal.pone.0239439)
Supplement: Attachment — Submitted filename: For reviewer.docx [file pone.0239439.s003.docx]

**RESPONSES TO COMMENTS**

We would like to thank the reviewers for their insightful comments, which have helped us to improve our paper.

**Reviewer 1**

***Comment 1:***	*In page 10 line 14, criteria of high linkage disequilibrium should be defined.*

**Response 1:**	Thank you for highlighting this. Accordingly, we have added the following text to the Materials and Methods section of the revised manuscript:

“High linkage disequilibrium was defined as r2 > 0.7.”

***Comment 2:***	*In the discussion, the authors should add a paragraph about the non-significant SNPs and their role, relationship to the topic.*

**Response 2:**	In accordance with the reviewer’s comment, we have added the following text to the Discussion section of the revised manuscript:

“*INHBA* rs2237432 is an intronic SNP that is classified as a synonymous SNP. Generally, non-synonymous SNPs are considered to affect gene behavior even more considerably than synonymous SNPs. However, some intronic SNPs may affect gene splicing or expression, and such SNPs may have an effect on the function of a gene [32,33]. Indeed, prediction tools revealed that *INHBA* rs2237432 might have a role as a strong enhancer of *INHBA* expression [33]. These suggest that rs2237432 is associated with the expression of *INHBA*.”

***Comment 3:***	*The authors should correlate their findings to the clinical setting more clearly. They should write about the application of their findings and how will it improve the follow up of the mCRC patients treated with regorafenib.*

**Response 3:**	Thank you very much for your comments. To address this, we have added the following text to the Discussion:

“A recent retrospective study showed comparable efficacy between regorafenib and TAS-102 [42]. However, a systemic review demonstrated that regorafenib was associated with more toxicity compared with TAS-102 [43]. On the basis of these results, female patients with the *INHBA* rs2237432 A/A allele should avoid regorafenib treatment and be treated with TAS-102 or best supportive care. Such a biomarker-based strategy will identify patients who are eligible for regorafenib treatment, resulting in improved clinical outcomes and quality of life for all patients treated with regorafenib.”

***Comment 4:***	*I see that the discussion don't include a relation of the significant findings with the mechanism of action of regorafenib.*

**Response 4:**	Thank you for pointing this out. In accordance with this comment, we have added the following text to the Discussion:

“Regorafenib is a small molecule that inhibits various intracellular kinases involved in tumor angiogenesis, metastasis, oncogenesis, and tumor immunity. Our results especially found a correlation with tumor angiogenesis. Regorafenib inhibits tumor angiogenesis through inhibiting VEGFR1-3 and TIE2. A preclinical study indicated that *INHBA* exerts diverse effects on the VEGF pathway, including upregulation of the ligand, VEGF, as well as VEGF receptors [16]. Considering these data together, *INHBA* polymorphism may be associated with the effect of regorafenib through exerting its actions via VEGFR.”

**Reviewer 2**

***Comment 1:***	*Where in the paper the authors discuss the control cohort of 128 patients receiving TAS-102? Did the authors compare the results of regorafenib to that of control cohort group?*

**Response 1:**	Apologies for our oversight. In accordance with the reviewer’s comment, we have added the following text to the Discussion:

“We included 128 patients who were treated with TAS-102 as the control cohort. TAS-102 is an oral drug that combines trifluridine and thymidine phosphorylase inhibitor [40]. The main antitumor effect of TAS-102 is due to DNA dysfunction by trifluridine incorporation into DNA [41]. In the TAS-102 cohort, contrary to the regorafenib cohort, *INHBA* rs2237432 at any G allele showed a trend toward worse PFS and OS compared with that at the A/A allele. However, these differences did not reach statistical significance, indicating that *INHBA* rs2237432 has a specific association with regorafenib efficacy. ”

***Comment 2:***	*One recent report suggests “regorafenib was associated with more toxicity compared with TAS-102” (Abrahao et al; Clin Colorectal Cancer. 2018 Jun;17(2):113-120). How this correlate with the current analysis? Authors should discuss this in detail.*

**Response 2:**	In accordance with the reviewer’s comment, we have added the following text to the Discussion:

“A recent retrospective study showed comparable efficacy between regorafenib and TAS-102 [42]. However, a systemic review demonstrated that regorafenib was associated with more toxicity compared with TAS-102 [43]. On the basis of these results, female patients with the *INHBA* rs2237432 A/A allele should avoid regorafenib treatment and be treated with TAS-102 or best supportive care. ”

***Comment 3:***	*Most of the correlations obtained from discovery cohort was opposite to the results obtained from validation cohort? Why the authors think this is happening? Smaller sample size? Different geographical condition? Authors should discuss this in detail.*

**Response 3:**	Thank you for your comment. We stated in the manuscript: “This finding may result from etiological differences between Japanese and Italian populations.” We have added the following text to the Discussion of the revised manuscript:

“The pathogenesis of cachexia may be influenced by various factors, including genetic predisposition, inflammatory cytokines, and hormonal aspects. Especially, SNPs within inflammatory cytokine genes can affect cytokine levels and the degree of inflammation, and these SNP functions are reported to differ according to ethnicity [49]”

***Comment 4:***	*Page 25, authors state “We analyzed data from 233 patients receiving regorafenib treatment in two cohorts”. However according to methods (page 6-7) it should be 230; which is correct?*

**Response 4:**	Thank you for highlighting our mistake. We have corrected the patient number to 230 in the revised manuscript.

***Comment 5:***	*Authors state “estrogen is reported to suppress activin subunit gene promoter activities, suggesting that activin activities differ by gender. These results may explain why the association between activin polymorphism and clinical outcome was dependent on gender”. This is interesting but this should be validated with the correlative analysis by comparing estrogen level in the patient group.*

**Response 5:**	We agree that additional information on serum or tissue biopsy would be valuable. Regrettably, however, because of the lack of samples, we are unable to undertake this experiment. We have added the following text to the Discussion section:

“Unfortunately, however, because of the lack of samples, we were unable to evaluate estrogen levels.”

***Comment 6:***	*Some validation data in patient’s serum or tissue biopsy would strengthen the impact of the study.*

**Response 6:**	Thank you for your suggestion. However, because of the lack of samples, we are unable to perform this experiment.

**Reviewer 3**

***Comment 1:***	*The authors also include many of the important references in the field including other clinical trials, however however they may wish to consider Abrahao et al., 2018 Clin. Colorectal Cancer 17(2):e113-20; Clarke et al., 2019 Cancer Chemother Pharmacol. 84(4):909-17; Fan et al., 2016 Oncotarget 7(39):64136-47; Ricci et al., 2020 World J. Gastrointest. Oncol. 12(3):301-310) that have examined regorafenib and various cytokines like TGFß, TNFα, VEGF, CCL2, CCL5 and CCL4.*

**Response 1:**	Thank you for your suggestion. We have added a citation in accordance with your suggestion.

43.	Abrahao ABK, Ko YJ, Berry S, Chan KKW. A Comparison of Regorafenib and TAS-102 for Metastatic Colorectal Cancer: A Systematic Review and Network Meta-analysis. Clin Colorectal Cancer. 2018;17: 113–120. doi:10.1016/j.clcc.2017.10.016
